# Rotational stability and refractive outcomes of a new hydrophobic acrylic toric intraocular lens

**DOI:** 10.1186/s40662-024-00393-2

**Published:** 2024-07-02

**Authors:** Daniel Schartmüller, Marcus Lisy, Nikolaus Mahnert, Markus Schranz, Victor Danzinger, Luca Schwarzenbacher, Stefan Pieh, Claudette Abela-Formanek, Christina Leydolt, Rupert Menapace

**Affiliations:** Department of Ophthalmology and Optometry of the Medical University of Vienna, Waehringer Guertel 18-20, 1090 Vienna, Austria

**Keywords:** Toric intraocular lens, Rotational stability, Refractive outcomes

## Abstract

**Purpose:**

To assess rotational stability and refractive outcomes of a new toric hydrophobic acrylic intraocular lens (IOL).

**Design:**

Single-center, prospective, interventional clinical trial.

**Methods:**

A total of 130 eyes of 82 patients with age-related cataract and total corneal astigmatism of greater than 1.0 diopters (D) received a hydrophobic acrylic toric IOL Clareon CNW0T3-9. Baseline measurement for rotational stability evaluation was performed at the end of surgery (EOS), with the patient still supine on the operating table, using non-movable vessels as reference landmarks. Postoperative retroillumination pictures were taken at 1 h, 1 week, 1 month and 4–6 months postoperatively. Subjective manifest refraction was assessed at the 6 months follow-up visit.

**Results:**

Final results were obtained in 126 eyes of 80 patients. Mean absolute rotation from EOS to 6 months was 1.33 ± 2.00 [0.01, 19.80] degrees. Rotational stability values from EOS to 1 h, 1 h to 1 week, 1 week to 1 month and 1 month to 6 months were 0.86 ± 0.82 [0.00, 3.90], 1.06 ± 1.94 [0.00, 19.45], 0.47 ± 0.42 [0.00, 2.03] and 0.38 ± 0.40 [0.00, 2.56] degrees. Mean preoperative corneal astigmatism was 1.78 ± 0.83 [1.00, 4.76] D which changed to a mean postoperative refractive astigmatism of 0.33 ± 0.27 [0.00, 1.25] D at 6 months.

**Conclusion:**

The Clareon toric IOL presented very good rotational stability with a mean absolute rotation below 1.4° from EOS to 6 months. Only two IOLs rotated more than 5° with none of them requiring repositioning surgery. Refractive outcomes were satisfying with a mean residual refractive astigmatism below 0.50 D.

**Trial registration:**

Registered at Clinicaltrials.gov NCT03803852; on May 17, 2022.

## Background

With the introduction of toric intraocular lenses (TIOL), a viable treatment for astigmatic corneal preconditions at the time of cataract surgery has been established [1]. Today, astigmatic errors of as little as 0.7 diopters (D) can be treated with the least powerful TIOLs available. While arcuate incisions, performed either manually or with femtosecond lasers, can treat low to moderate astigmatic errors, only TIOLs should be used to treat higher astigmatic errors [2–5]. The key factors for the treatment of astigmatism at the time of cataract surgery are precise preoperative diagnostics, exact intraoperative alignment, and excellent rotational stability of the TIOL. Preoperative topographic and tomographic measurements may vary across different devices. While topographic measurements exclude posterior astigmatism, tomographic measurements provide values for total corneal astigmatism.

The stability of TIOLs relies on multiple factors. The rotational stability of C-loop haptic IOLs is influenced by factors such as different materials, varied haptic configurations, and the overall diameter [6]. Several approaches have been used to prevent postoperative IOL rotation. These approaches include simultaneous implantation of capsular tension rings (CTR), suturing the TIOL to the CTR, or transscleral suturing of postoperatively rotated TIOLs [7–10].

The AcrySof IQ IOL (Alcon, Fort Worth, TX), a predecessor to the Clareon TIOL (Alcon, Fort Worth, TX), has already shown very good rotational stability in various studies [6, 11]. The Clareon TIOL shares the same haptic design and overall diameter as the AcrySof TIOL. However, variations in the material used may affect the haptic reset force or surface characteristics, which can impair the IOLs rotational stability.

Accordingly, this study examines the rotational stability and refractive outcomes over a period spanning from the end of surgery (EOS) to 6 months postoperatively using a high precision evaluation method.

## Methods

This single-center, multi-surgeon, prospective, clinical trial was conducted at the Medical University of Vienna. A total of 130 eyes of 82 patients were included in the study. Surgeries were conducted between June 2022 and March 2023. All surgeries were performed by five highly experienced cataract surgeons (D.S., C.L., R.M., C.A-F., M.S.). All study procedures adhered to the Tenets of the Declaration of Helsinki. The study was approved by the local ethics committee (EK 1978/2018) and registered at a public clinical trial registry (NCT03803852) prior to the beginning of the study. All patients provided written informed consent prior to study inclusion.

Inclusion criteria were total corneal astigmatism of 1.00 D or more measured by anterior-segment optical coherence tomography (AS-OCT) in combination with a Placido disc (MS-39, CSO, Firenze, Italy), uni- or bilateral age-related cataract, age from 45 to 95 years, need for a spherical IOL between 6.00 and 30.00 D and a preoperative pupil width of at least 5.5 mm.

Exclusion criteria were previous ocular surgeries or ocular trauma, blind fellow eye, history of uveitis, expected zonular weakness, proliferative diabetic retinopathy, uncontrolled glaucoma, pregnancy, corneal abnormalities such as corneal scars and other uncontrolled ocular or systemic diseases. Patients with pseudoexfoliation syndrome or diffuse zonular weakness at the time of surgery without the need for a CTR were not excluded. Primary outcome measures were absolute postoperative TIOL rotation from EOS to 4–6 months and the refractive outcome for each patient.

### Preoperative examinations

One to two weeks prior to surgery, preoperative diagnostics were performed. Preoperative examinations included visual acuity by an autorefractor keratometer Nidek ARK-1 (Nidek Co Ltd, Tokyo, Japan), biometry using an IOL Master 700 (Carl Zeiss Meditec AG, Jena, Germany), AS-OCT combined with Placido tomography MS-39 (CSO, Firenze, Italy), AS-OCT Casia 2 (Tomey Corporation, Nagoya, Japan) under miotic and mydriatic pupil conditions, standard macular OCT measurements with a high-definition optical coherence tomography (HD-OCT) Cirrus 6000 (Carl Zeiss Meditec, Jena, Germany) and a standard slit-lamp examination. Aqueous depth, lens thickness and lens equatorial diameter were obtained with the AS-OCT under mydriatic conditions. Axial length (AXL) and anterior chamber depth were obtained with the IOL Master 700.

### Toric IOL calculation

The spherical equivalent (SE) of the TIOL was determined using an IOL Master 700 print using a “Mean Formula” between Barrett TK Universal II (total keratometry) and the Haigis (with TK values) formulae. Due to the novelty of the TIOL, no optimized A-constants were available at the time of study inclusion and therefore a mean of these two institutional used formulae was used.

The magnitude of the cylinder for the TIOL was calculated in the Alcon online toric calculator (Holladay formula) using the refractive analysis values of the MS-39 AS-OCT (K-Index 1.3375) within the 4.5 mm optical zone (total keratometry including the backside of the cornea). The surgically induced astigmatism (SIA) was set to zero. The TIOL was implanted to the steep axis (Ks) of the MS-39 AS-OCT refractive analysis measurement.

### The IOL

The Clareon TIOL has an overall diameter of 13.0 mm and an optic diameter of 6.0 mm with no haptic angulation. It features the identical design, meaning the same mechanical and optical attributes, as its predecessor lens, the well-established AcrySof IQ TIOL. The AcrySof TIOL is also popular for its bioadhesive material, providing good rotational stability in different studies [12]. The new IOL contains hydroxyethyl methacrylate instead of phenylethyl methacrylate aimed to eliminate glistenings and surface light scattering [13]. The Clareon TIOL is available from 6.00 to 30.00 D in SE and from 1.00 to 6.00 D (T2 = 1.00 D, T3 = 1.50 D, T4 = 2.25 D, T5 = 3.00 D, T6 = 3.75 D, T7 = 4.50 D, T8 = 5.25 D, T9 = 6.00 D) corrective cylindric power at the IOL plane. The IOL has a negative spherical aberration of 0.2 µm.

### Surgery

All surgeries were performed using a Lumera 700 (Carl Zeiss Meditec AG, Jena, Germany) surgical microscope. Prior to the surgery, the scleral reference picture of each patient from the IOL Master 700 was imported to the Callisto System. Two safety marks on the limbus were done at the planned steep axis using a surgical pen after synchronizing the reference picture with the actual picture of the surgical microscope. A temporal posterolimbal corneal incision of 2.2 to 2.4 mm was performed in all cases. According to the surgeon’s preference, one to two side ports approximately 45° apart from the posterolimbal corneal incision were created. After an approximately 5.0 to 5.5 mm capsulorhexis, nucleus removal, coaxial irrigation and aspiration of the remaining cortex were performed. For implantation of the TIOL, strictly cohesive ophthalmic viscoelastic device (OVD) only was used (Provisc, Alcon, Fort Worth, TX). After rotating the IOL to the planned axis, thoroughly the OVD was removed with special attention to the retrolental space and capsular bag fornix. After stromal hydration of the wounds, intracameral antibiotic was injected (Cefuroxime 1 mg/0.1mL). Again, the TIOL axis was checked and verified with the Callisto marking system and the axis markings on the limbus.

To eliminate measurement errors, a video clip was recorded immediately after wound closure at the EOS. This clip compared the actual axis of the IOL to scleral landmarks, using a method previously described by our study group [14]. In short, a surgical swab was used to move the conjunctiva to distinguish between movable conjunctival and non-movable scleral and episcleral landmarks. These landmarks were used to compare the actual IOL axis at the EOS to the IOL axis at follow-up visits at 1 h, 1 week, 1 month and 4–6 months.

### Follow-up visits and axis determination

At every follow-up visit, a retroillumination picture using a high-definition digital camera DCS720x (Kodak, Rochester, New York, USA) was performed. These pictures were imported into the semi-automated evaluation software Rotix [14]. Two lines were drawn to determine the IOL axis, one between two non-movable vessels serving as the reference axis and a second one between the axis markings of the IOL. The Rotix software then automatically calculates the axis difference between follow-up visits. At the 6 months follow-up visit, subjective refraction and vision testing were performed using a Snellen chart at 6 m distance applying the cross-cylinder method. To determine the SIA, a total keratometry (TK), using the postoperative cataract module of the AS-OCT, was performed at 6 months.

### Sample size

According to current ISO and American National Institute Standards (ANSI), at least 100 IOLs should be evaluated to assess the rotational stability of a TIOL. From earlier studies, we know that between 20% and 25% of patients were either lost to follow-up or not evaluable for the primary outcome due to non-visibility of landmarks at the sclera at the EOS or at follow-ups. Therefore, a sample size of 130 eyes was calculated.

### Statistical analysis

Explorative data analysis was performed for rotational stability measurements at all time points. Rotational stability data is presented as mean ± standard deviation and as median [range] in absolute values. Preoperative corneal astigmatism, postoperative refractive astigmatism, and SIA are presented in double-angle plots recently developed by Abulafia et al. [15]. As a secondary objective correlation between AXL, lens thickness and lens equatorial diameter were computed using Spearman’s rho. The binominal test was used to calculate the difference in the direction of rotation between EOS and 6 months. *P* values less than 0.05 were considered statistically significant throughout, and no multiplicity correction was applied.

## Results

One-hundred thirty eyes of 82 patients were included in this study. Among the 130 eyes, 72 (55%) were right eyes and 58 (45%) were left eyes. Of the 82 patients, 42 (51%) were women and 40 (49%) were men. Thirty-four patients were treated unilateral, and 48 patients were treated bilateral with TIOL implantation. Of all 130 eyes, 53 (40.8%) eyes had with-the-rule corneal astigmatism (WTR = 67.5° to 112.5°), 58 (44.6%) eyes had against-the-rule corneal astigmatism (ATR = 0° to 22.5° and 157.5° to 180°), and 19 (14.6%) eyes had oblique corneal astigmatism (Oblique = 22.5° to 67.5° and 112.5° to 157.5°). Table 1 gives an overview of patients demographics and descriptive statistics.

**Table 1:** Patients demographics preoperatively

Parameters	Mean ± SD [min, max]
Age (years)	71.5 ± 9.0 [49, 89]
Sphere (D)	− 2.20 ± 3.84 [− 17.50, 7.00]
Keratometric cylinder (D)	1.78 ± 0.83 [1.00, 4.76]
Spherical equivalent (D)	− 1.20 ± 3.50 [− 15.25, 7.50]
Axial eye length (mm)	23.8 ± 1.3 [20.9, 28.4]
K1 (D)	43.0 ± 1.6 [39.8, 47.1]
K2 (D)	44.7 ± 1.7 [41.5, 49.4]
AQD (mm)	2.7 ± 0.4 [1.9, 4]
Lens thickness (mm)	4.7 ± 0.4 [3.9, 5.7]
Lens equatorial diameter (mm)	10.2 ± 0.5 [8.7, 12.0]
Postoperative target refraction^a^ (D)	− 0.45 ± 0.22 [− 1.12, 0.175]

Keratometric Index of the measurement device was 1.3375

*K1* = flat keratometry; *K2* = steep flat keratometry; *AQD* = aqueous depth; *SD* = standard deviation

^a^Excluding patients targeted for myopia

### Rotational stability

**Fig. 1:**
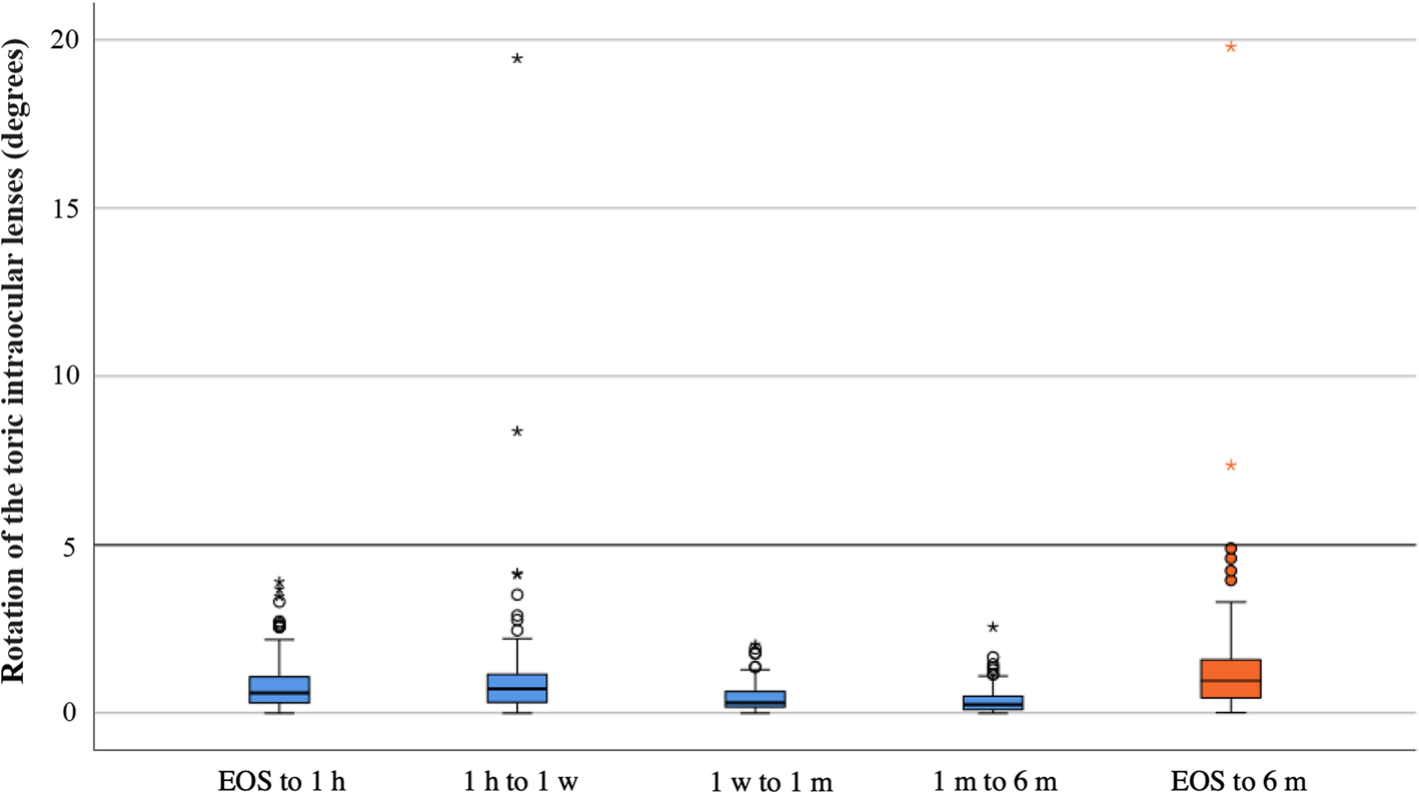
Rotation of the toric intraocular lenses from the end of surgery to 6 months after surgery (orange) and between the individual follow-up visits (blue). EOS, end of surgery; h, hour; m, month

For the analysis of the rotational stability of the Clareon TIOL, 125 (96.2%) TIOLs could be evaluated for the main outcome, the rotation from EOS to 6 months. All 130 eyes underwent surgery with TIOL implantation. Four eyes (from two patients) were lost to follow-up, and one eye could not be analyzed due to insufficient episcleral landmark visibility. Detailed results of the rotational stability from EOS to 1 h, 1 h to 1 week, 1 week to 1 months, 1 month to 6 months and EOS to 6 months are shown in Table 2 and Fig. 1.

**Table 2:** Mean and median for intraocular lens (IOL) rotation and its outliers with IOL rotation of more > 5°, > 10° and > 15°

Time	No. of eyes	Median [range]	Mean ± SD	IOLs rotating more than		
				** > 5°** **n (%)**	** > 10°** **n (%)**	** > 15°** **n (%)**
**EoS to 1 h**	127	0.60 [0.00, 3.90]	0.86 ± 0.82	0	0	0
**1 h to 1 w**	125	0.72 [0.00, 19.45]	1.06 ± 1.94	2 (1.6%)	1 (0.8%)	1 (0.8%)
**1 w to 1 m**	122	0.31 [0.00, 2.03]	0.47 ± 0.42	0	0	0
**1 m to 6 m**	122	0.25 [0.00, 2.56]	0.38 ± 0.40	0	0	0
**EoS to 6 m**	125	0.96 [0.01, 19.80]	1.33 ± 1.99	2 (1.6%)	1 (0.8%)	1 (0.8%)

*SD* = standard deviation; *EoS* = end of surgery; *h* = hour; *w* = week; *m* = month

Over the 6-month follow-up period, 95 TIOLs rotated clockwise, and 29 TIOLs rotated counterclockwise (*P* < 0.01). One TIOL showed zero rotation.

### Refractive outcomes

**Fig. 2:**
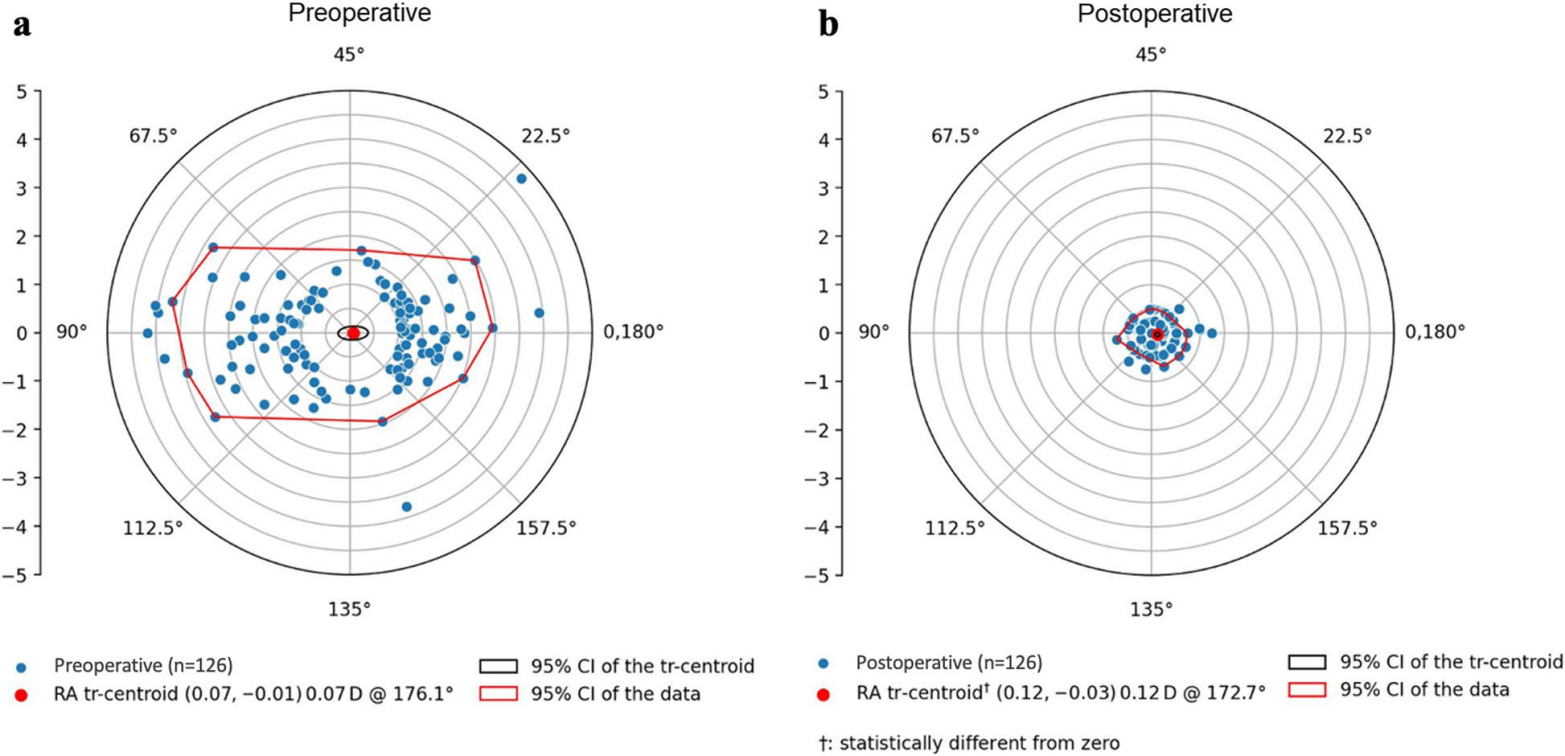
Comparison of preoperative corneal astigmatism (**a**) and postoperative refractive astigmatism (**b**) at the corneal plane in a double-angle plot. A clear centralization of the vectors around the center can be seen postoperatively. The centroid of the postoperative refractive astigmatism is statistically different from zero

**Fig. 3:**
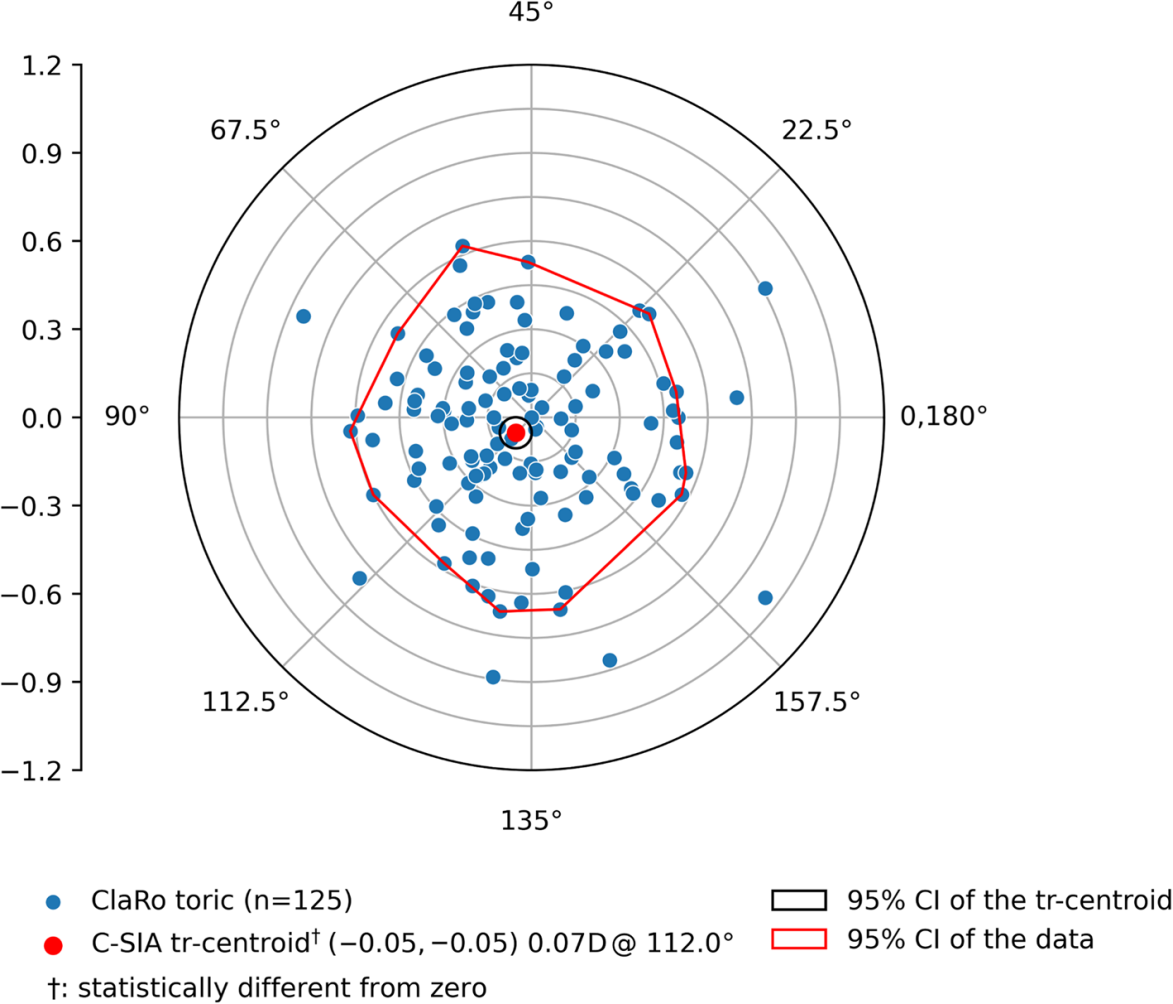
Double-angle plot of the surgically induced astigmatism

The preoperative corneal astigmatism and the postoperative refractive astigmatism are shown as double angle plots in Fig. 2. The SIA is shown as double angle plot in Fig. 3 according to a new illustration by Abulafia, Kan-Tor and Benjamini – Eyetamis analysis tool (www.eyetamis.com, accessed 14.11.2023). Preoperative corneal astigmatism was 1.78 ± 0.83 [1.00, 4.76] D and postoperative refractive astigmatism was 0.33 ± 0.27 [0.00, 1.25] D. Mean SIA was 0.38 ± 0.20 [0.00, 1.00] D with a centroid of 0.07 D at 112.0°.

**Fig. 4:**
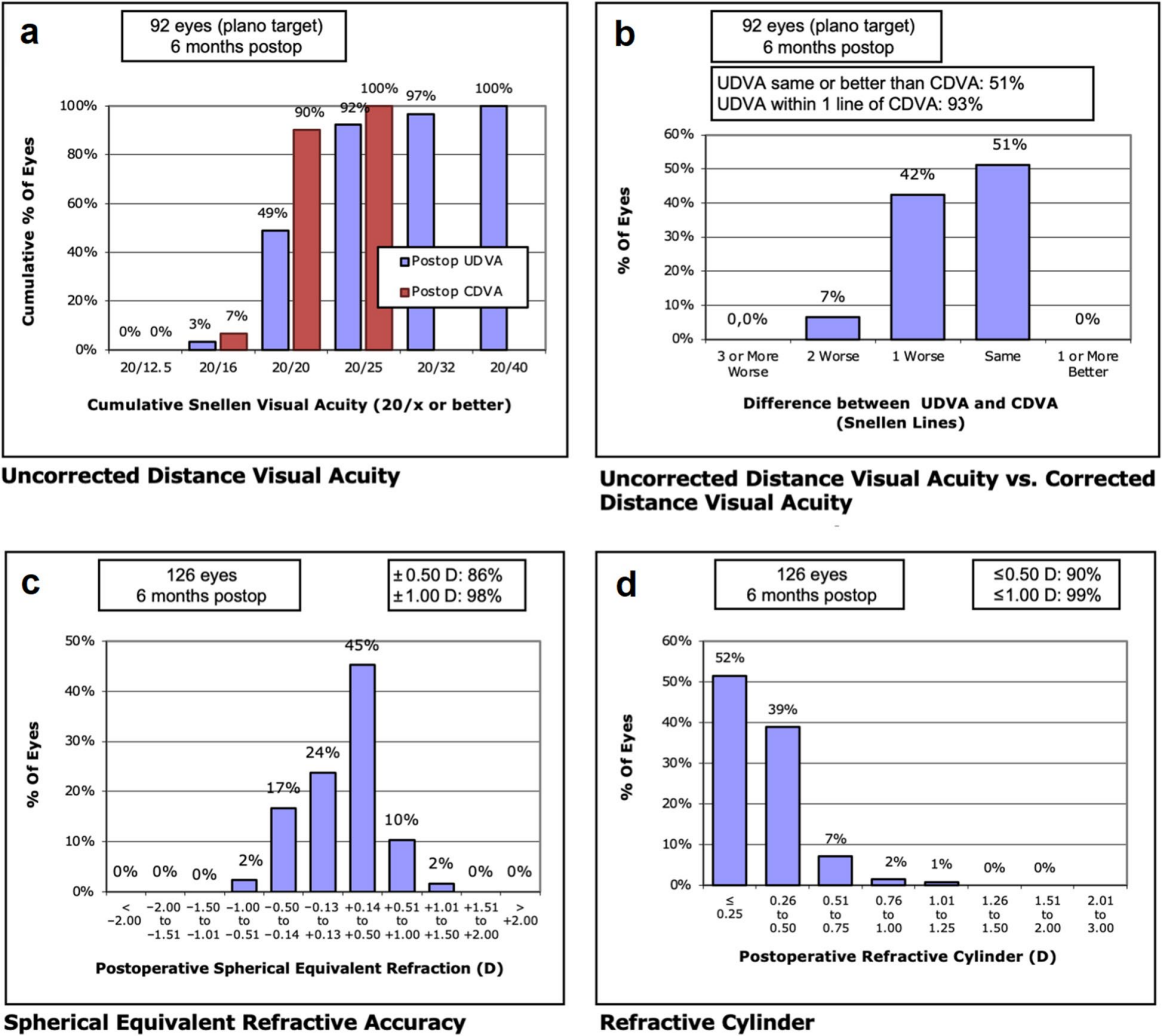
The standard graphs for reporting refractive outcomes for intraocular lens-based procedures in a cataract population. **a** Corrected and uncorrected visual acuity of all patients within ± 0.50 D spherical equivalent postoperatively. Figure 4a shows the cumulative corrected and uncorrected distance visual acuity for all eyes targeted for plano. **b** Uncorrected distance visual acuity versus corrected distance visual acuity. Figure 4b shows percentage of eyes having the same or worse uncorrected distance visual acuity in comparison to corrected distance visual acuity. **c** Spherical equivalent refraction accuracy. Figure 4c shows the spherical equivalent refraction accuracy for all eyes. A slightly hyperopic shift was observed. **d** Distribution of the postoperative refractive cylinder. Figure 4d shows the refractive cylinder achieved postoperatively. Ninety percent of eyes achieved a residual postoperative refractive cylinder within 0.50 D and 99% of eyes achieved a residual postoperative refractive cylinder within 1.00 D

The visual and refractive accuracy outcomes are shown in Fig. 4 according to a method published by Reinstein et al. [16]. The prediction error (PE) and absolute prediction error (A-PE) was calculated for the Barrett, the Haigis and the “Mean Formula” (mean between Barrett and Haigis, used due to lack of optimized A-constants at the time of the study). The PE for the Barrett, Haigis and “Mean Formula” was 0.23 ± 0.33 D, 0.11 ± 0.35 D and 0.17 ± 0.32 D. The A-PE for the Barett, Haigis and “Mean Formula” was 0.32 ± 0.23 D, 0.29 ± 0.22 D and 0.29 ± 0.21 D.

In total, 55 (42%) T3, 33 (25%) T4, 17 (13%) T5, 10 (8%) T6, 4 (3%) T7, 7 (5%) T8 and 4 (3%) T9 TIOLs were implanted.

Preoperatively, 75 (58%) out of 130 eyes were targeted for plano (± 0.50 D) whereas 41 eyes (32%) where targeted between − 0.50 D and − 0.75 D. Due to a small hyperopic shift in the refractive outcomes, more IOLs than targeted achieved a plano outcome. In total, 95 (75.4%) of the 126 eyes available for the 6 months follow-up achieved a postoperative SE of plano ± 0.50 D. Of these 95 eyes, three eyes were excluded from visual acuity evaluations. One eye was excluded due to a preoperatively unnoticed nutritional toxic alcoholic ganglion cell layer thinning, and two eyes were excluded due to postoperatively detected amblyopia (two different patients with manifest hyperopia at the time of surgery).

### Correlations

There was no correlation between the preoperative AXL and rotation from EOS to 6 months (*r* = 0.11, *P* = 0.21), no correlation between the crystalline lens thickness and rotation from EOS to 6 months (*r* = 0.03, *P* = 0.76) and no correlation between crystalline lens equatorial diameter and rotation from EOS to 6m (*r* = 0.04, *P* = 1.0).

No serious adverse events were observed during the course of the study.

## Discussion

The success of a TIOL depends on several different factors. In addition to precise preoperative measurements, accurate calculation, and right placement at the time of surgery, the rotational stability is one of the key factors of a TIOL. Postoperative IOL rotation often results in residual astigmatism, which can sometimes lead to secondary re-rotation procedures. These surgeries do not only bring financial burden to the patient and/or the public healthcare system and pose serious risks such as zonular stress and dehiscence [17, 18], recurrent re-rotation, additional endothelial cell loss, endophthalmitis and all other risks of an intraocular intervention. Therefore, a surgeon might choose a TIOL with the lowest possible rotation reported. To our knowledge, this is the first study reporting rotational stability and refractive outcomes of the Clareon TIOL.

First, to assess the rotational stability of a toric IOL, a method, independent of head tilt, cyclotorsion as well as autorotation [19] using reference landmarks at the sclera should be employed. Second, baseline measurement should be performed immediately at the EOS to assess the true IOL axis at the EOS [20]. An initial offset to the implantation axis has been shown in an earlier study, this offset could contribute to or even equalize postoperative rotational stability measurements [21]. Furthermore, it has been reported that postoperative rotation mostly occurs within the very first hour after surgery [20]. As a result, evaluating the baseline axis after the surgery may lead to erroneous results.

**Fig. 5:**
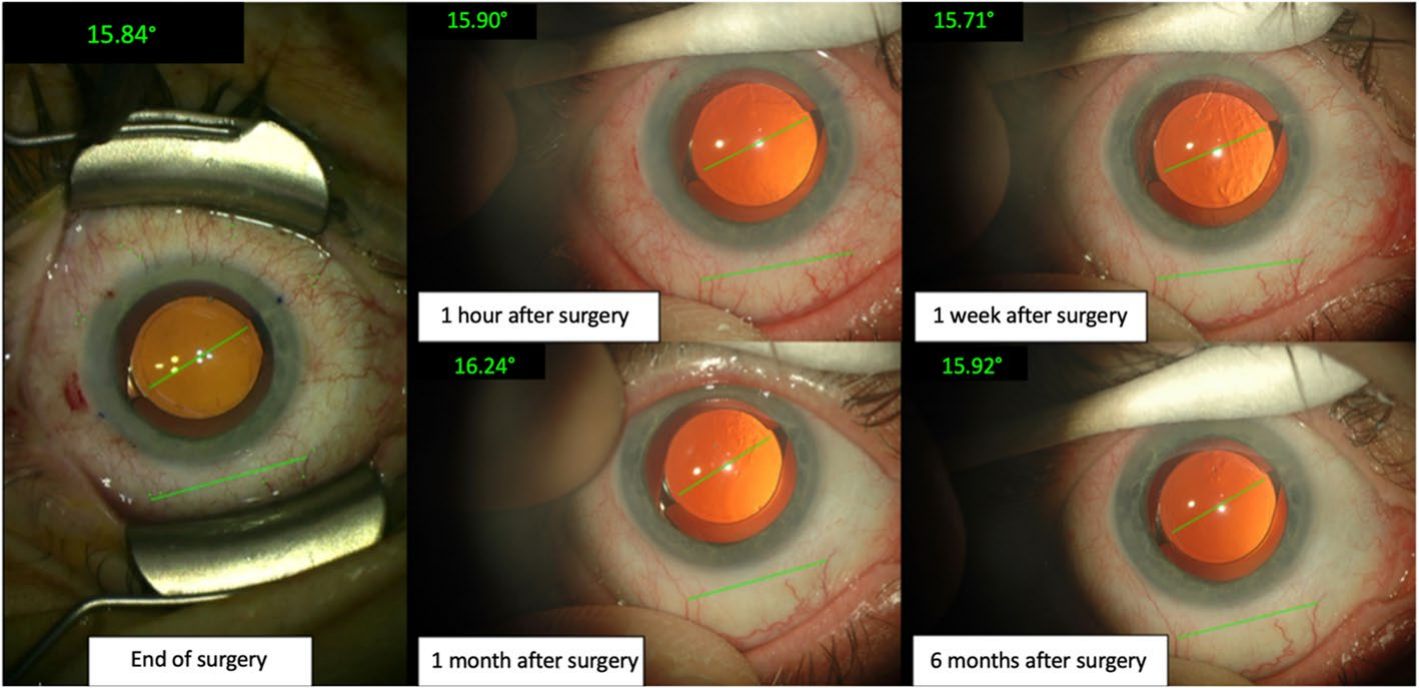
An image series of the rotational stability evaluation on a right eye with a total rotation of only 0.08° after 6 months postoperatively

**Fig. 6:**
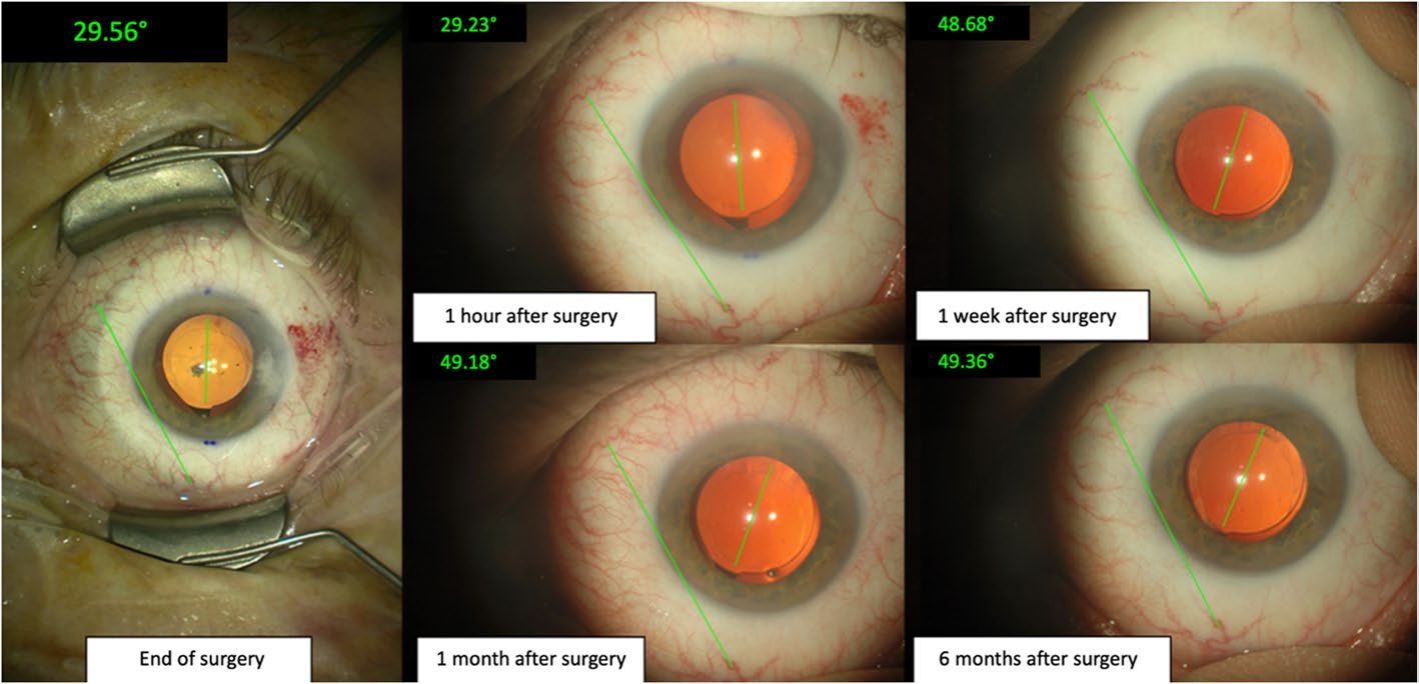
An image series of the rotational stability evaluation on a left eye with a rotation of 19.45° between 1 h and 1 week after the end of surgery

The Clareon TIOL in our study showed a mean absolute rotation of 1.33 ± 1.99° with an even lower median of 0.96° from EOS to 6 months postoperatively. In 126 eyes, only two IOLs rotated more than 5° from its initial axis at the EOS. One IOL (a T3 toric IOL) rotated 7.4° from EOS to 6 months leading to a residual astigmatism of 0.50 D. The second IOL rotated 19.8° from EOS to 6 months (T3) IOL leading to a residual astigmatism of 0.50 D. In both patients, no repositioning surgery was necessary due to the low amount of residual astigmatism. However, with increasing cylindric power of the TIOL, the sensitivity to postoperative IOL rotation is also increasing, and thus a possible re-rotation surgery could not be ruled out in case of a higher toric power IOL in these cases. Figures 5 and 6 show the evaluation of two TIOLs with baseline photography at the table and subsequent retroillumination photography at follow-ups – one IOL rotating 0.08° from EOS to 6 months and the major outlier exhibiting 19.8° offset were observed.

The widely used “rule-of-thumb” of 3% corrective effect loss for every degree of misalignment might not apply to all TIOLs [1]. A recent study by Tognetto et al. found that postoperative rotation of 30° reduces the image quality by less than 50% [22]. However, the image quality always depends on the toric power of the TIOL, and thus may be more compromising in higher power TIOLs.

Results from this study indicate that the rotational stability of the Clareon TIOL is consistent with its predecessor, the Acrysof IOL, demonstrating very good postoperative rotational stability. In a recent study, our research group compared the rotational stability of the non-toric Acrysof IOL, Tecnis ZCB00 IOL, and Envista IOL utilizing the same methodology [6]. The Acrysof IOL showed postoperative rotation of median 1.1° and a mean of 1.65 ± 2.1° which is almost identical to the results demonstrated in this study. A higher proportion of outliers of more than 5° was observed with the Acrysof IOL than with the Clareon TIOL in the present study (4.8% vs*.* 1.6%). Differences in surface coating material may contribute to the observed improvement in rotational stability. The Acrysof IOL is known for their high bioadhesivity [12]. From a clinical perspective, it is confirmed that the Clareon TIOL features similar characteristics. The Clareon TIOL presented challenges in making small adjustments at the EOS when the OVD has already been removed, which could be due to high bioadhesivity of the TIOL surface.

Recent studies demonstrated that the majority of IOL rotation occurs within the initial hour following surgery [6, 20, 21, 23]. Interestingly, both TIOLs that rotated more than 5° in our study rotated beyond 1 h post-surgery. This could be explained as follows: In the present study, it was observed that the majority IOLs were initially easy to rotate within the capsular bag up to a specific point before getting stuck. This phenomenon could be attributed to the elliptical shape of the capsular bag [24]. Hypothetically, in the present two cases of postoperative rotation, the lens may have been positioned directly at the edge of the largest diametrical expansion of the capsular bag equator. The consolidation of the capsular bag during the first week might have caused the IOL to rotate towards the largest diametral spread of the bag.

A recent study evaluated the rotational stability of the Tecnis Eyhance DIU TIOL on the Tecnis toric II platform [25]. In contrast to their predecessor lens, the Tecnis toric II IOL features frosted haptic edges which were first introduced with the Vivinex IOL (Hoya, Nagoya, Japan) [20]. In this study evaluating the Tecnis DIU TIOL, the authors found a mean IOL rotation of 1.35 ± 1.46° which is similar to the results in our study. The authors found a maximum rotation of 6.2°. However, only 27 out of 50 IOLs could be evaluated due to insufficient pupil mydriasis or loss to follow up postoperatively. In the present study, the first outlier was identified with patient ID number 66. Certainly, this outlier would have gone unnoticed if we had not aimed for a proper sample size of at least 100 evaluable TIOLs, as recommended by the International Organization for Standardization (ISO) and the American National Standards Institute (ANSI) guidelines [26, 27].

In a retrospective cohort study, Lee and Chang investigated the repositioning rates of the Acrysof and Tecnis TIOLs [11]. For the Acrysof TIOL, the authors found a likelihood for a repositioning surgery of 1.6% in 626 eyes for the Acrysof TIOL and 3.1% for the Tecnis TIOL (based on the Tecnis I IOL). In our cohort, we would only expect one repositioning surgery if in this case a higher cylindrical power TIOL would have been used, which corresponds to 0.8%.

It has been demonstrated that including posterior astigmatism in TIOL calculation improves postoperative refractive outcomes [28, 29]. The posterior astigmatism can be addressed through nomograms for posterior astigmatism correction, such as Abulafia-Koch, Koch-Wang, Barrett, Baylor, or by directly measuring with tomographers that use Scheimpflug or OCT [30, 31]. A recent study has shown that strictly topographic measurements may not consider back surface astigmatism appropriately, and therefore lead to inaccuracies in TIOL calculation [32]. In the present study, we measured total corneal astigmatism by utilizing the total keratometry values of the MS-39 AS-OCT in the 4.5 mm zone leading to satisfying refractive results. In 90% of eyes, a postoperative refractive astigmatism of ≤ 0.50 D and in 99% of eyes ≤ 1.00 D could be achieved. In our study, the SIA was set to 0.00 D for the calculation of the toric IOL. Wendelstein et al. found a SIA of approximately 0.30 D (centroid) in post-cataract eyes when performing a superior limbal incision using a 2.5 mm approach [33]. Our study found a mean SIA of 0.38 D with a centroid of 0.07 D. The low centroid value of 0.07 D indicated that the small 2.2 to 2.4 mm temporal posterolimbal corneal incision has almost no systematic astigmatic effect. However, the mean SIA of 0.38 D, with some eyes showing a SIA of more than 0.50 D, suggest that changes in corneal astigmatism can be expected postoperatively.

Only one IOL showed a residual astigmatism of 1.25 D. In this case, an undercorrection of 0.49 D of an astigmatism against-the-rule was aimed and led to an undercorrection of 1.25 D on the same axis. Interestingly, the total corneal astigmatism against-the-rule was 0.50 D higher postoperatively even though a temporal 2.2 mm posterolimbal incision was performed (2.40 D preop vs*.* 2.90 D postop at 0°, Casia 2). Our study results indicate that the SE for the target refraction was slightly underestimated. The novel lens used in our study lacked optimized A-constants at the time of study conduction. Therefore, we utilized the mean between the Barret Universal II TK and the Haigis Formula for our target refraction.

In the past, discrepancies in the axis and power of TIOL calculations were observed when comparing measurements of total corneal astigmatism to calculations of posterior corneal astigmatism using regression formulae [30, 34, 35]. In our study, we employed the 4.5 mm refractive analysis of the MS39 tomographer, resulting in highly favorable postoperative refractive outcomes.

## Conclusions

To conclude, the Clareon toric intraocular lens demonstrated very high rotational stability, with only two IOLs rotating more than 5° from the EOS to 6 months with none of them requiring repositioning surgery. AXL, crystalline lens thickness and crystalline lens equatorial diameter were not correlated with TIOL rotation. Bioadhesive surface properties may account for good rotational stability despite lack of frosted haptics. The refractive outcomes were predictable, suggesting that utilizing the 4.5 mm zone of the MS39 OCT tomographer is a reliable technique for computing the toric power of a TIOL. The Clareon TIOL is a safe and effective treatment for astigmatism at the time of cataract surgery.

## Data Availability

The datasets used and/or analyzed during the current study are available from the corresponding author upon reasonable request.

## Abbreviations

AS-OCT: Anterior-segment optical coherence tomography
AXL: Axial length
CTR: Capsular tension ring
D: Diopter
EOS: End of surgery
LE-D: Lens equatorial diameter
PE: Prediction error
SE: Spherical equivalent
SIA: Surgically induced astigmatism
TIOL: Toric intraocular lens
